# Evaluation of Weight Gain and Overall Survival of Men Versus Women With Advanced Non‐Small Cell Lung Cancer

**DOI:** 10.1002/jcsm.70131

**Published:** 2025-12-03

**Authors:** Eric J. Roeland, Florian J. Fintelmann, Ruoyong Yang, Lisa Tarasenko, Philip D. Bonomi

**Affiliations:** ^1^ Knight Cancer Institute Oregon Health and Science University Portland Oregon USA; ^2^ Department of Radiology Massachusetts General Hospital Boston Massachusetts USA; ^3^ Pfizer Inc New York New York USA; ^4^ Rush University Medical Center Chicago Illinois USA

**Keywords:** cachexia, non‐small cell lung cancer, survival, weight, weight gain

## Abstract

**Background:**

Weight stabilization or gain during cancer treatment is associated with increased survival. Analyses of weight gain by sex during cancer treatment and the effects of weight on survival have not been fully studied. This post hoc analysis retrospectively examined the relationship between sex, weight gain and overall survival (OS) in patients with advanced non‐small cell lung cancer (NSCLC) receiving standard‐of‐care chemotherapy.

**Methods:**

Data from the control arms of three randomized phase III clinical studies (NCT00596830, NCT00254891, NCT00254904) of adult patients with advanced NSCLC were pooled; patients received platinum‐based doublet chemotherapy. Weight was recorded according to each study's schedule. Analyses compared weight gain categories from baseline up to 3 months after chemotherapy initiation (> 0% vs. ≤ 0%, > 2.5% vs. ≤ 2.5% and > 5% vs. ≤ 5%). Stepwise Cox proportional hazards modelling of OS (time from treatment initiation to death due to any cause) was used to estimate hazard ratios (HRs) with 95% confidence intervals (CIs); differences between groups were tested with log‐rank tests.

**Results:**

Of the 1030 patients, most were men (70.5%) with stage IV NSCLC. Median (range) age was 62 (34–87) years for men and 60 (34–83) years for women, and the mean body mass index (standard deviation) was 24.5 (4.2) and 24.8 kg/m^2^ (4.8), respectively. Weight gain of > 0%, > 2.5% and > 5% was observed in 44.0%, 24.5% and 11.7% of the patients, respectively. Any weight gain (> 0% vs. ≤ 0%) was associated with a significantly reduced risk of death in both men (HR 0.62 [95% CI 0.522, 0.742]; *p* < 0.0001) and women (HR 0.68 [95% CI 0.512, 0.910]; *p* = 0.0092). Weight gain of > 2.5% vs. ≤ 2.5% was associated with a significantly reduced risk of death in men (HR 0.64; 95% CI 0.516, 0.784; *p* < 0.0001) and a risk reduction in women (HR 0.73; 95% CI 0.518, 1.034; *p* = 0.0767). Weight gain of > 5% vs. ≤ 5% was associated with a significantly reduced risk of death in men (HR 0.67; 95% CI 0.503, 0.879; *p* = 0.0042), but not in women (HR 0.90; 95% CI 0.542, 1.509; *p* = 0.70). Despite these differences, the overall interaction of weight gain by sex was not significant (*p* = 0.61 for > 0% vs. ≤ 0%, *p* = 0.43 for > 2.5% vs. ≤ 2.5% and *p* = 0.37 for > 5% vs. ≤ 5%).

**Conclusions:**

Any weight gain during treatment of advanced NSCLC was associated with a significantly reduced risk of death, regardless of sex.

**Trial Registration:** NCT00596830; NCT00254891; NCT00254904.

## 1 Introduction

Cancer cachexia is a multifactorial syndrome characterized by anorexia, unintentional weight loss (adipose and skeletal muscle), fatigue, functional impairment, poor quality of life and decreased survival [1, 2, 3]. Equally, weight stabilization or weight gain during cancer treatment is associated with increased survival [4, 5, 6, 7, 8, 9]. In a recent analysis of data pooled from three clinical studies, the survival benefit was comparable for any weight gain across weight gain categories (i.e., > 0%, > 2.5%, > 5%), suggesting that any weight gain may be an early predictor of survival [9]. The likelihood and degree of weight gain during cancer treatment in men compared to women and the effects of weight gain on survival have not been fully studied [4, 5, 6, 7, 8, 9].

The objective of this post hoc analysis was to examine the relationship between sex, weight gain and overall survival (OS) in patients with advanced non‐small cell lung cancer (NSCLC) receiving standard‐of‐care chemotherapy.

## 2 Methods

### 2.1 Study Participants

Data were pooled from control arms of three multinational, open‐label, randomized, phase III clinical studies (NCT00596830, NCT00254891, NCT00254904) conducted between November 2005 and March 2011 in adult patients (age ≥ 18 years) with advanced NSCLC, similar to a previously published study [9]. Key inclusion criteria included a diagnosis of stage IIIB (with pleural effusion) or stage IV NSCLC; no previous systemic treatment for NSCLC with chemotherapy, immunotherapy or biologic response modifiers (NCT00596830 permitted chemotherapy completed ≥ 1 year before randomization); and Eastern Cooperative Oncology Group performance status of 0–1 [10]. Key exclusion criteria included central nervous system metastasis and a diagnosis of small cell or carcinoid lung cancer. The studies were conducted in accordance with the Declaration of Helsinki and all participants provided informed consent.

Patients in the control arms of the studies received first‐line standard platinum‐based doublet chemotherapy (paclitaxel and carboplatin in NCT00254891 and NCT00596830; gemcitabine and cisplatin in NCT00254904). A maximum of six 3‐week cycles of chemotherapy were administered.

### 2.2 Assessments and Data Analysis

Investigators recorded weight at baseline, before dosing on day 1 of each 3‐week treatment cycle (continued for up to 6 cycles), and after treatment according to each study's schedule. For each patient, the percent change in weight from baseline was calculated at all planned and unplanned visits up to 3 months after chemotherapy initiation, and the maximum percentage weight gain was determined. Aligned with the original studies, the analyses used patient subgroups based on maximum percentage weight gain categories of > 0% vs. ≤ 0%, > 2.5% vs. ≤ 2.5% and > 5% vs. ≤ 5% from baseline up to 3 months [9]. Analyses to determine the optimum weight gain cut‐off were not conducted, as this was not an objective of this post hoc analysis.

The Kaplan–Meier method was used to estimate OS and median OS by sex and weight gain. OS was defined as the time from treatment initiation to death due to any cause. Patients were censored on the date of last contact.

Stepwise Cox proportional hazards modelling of OS was used to estimate a hazard ratio (HR) with a 95% confidence interval (CI) between categories of weight gain. In the overall analysis, covariates included weight gain, study, sex, age, smoking status and interactions of weight gain by sex. In the analysis of the men and women subgroups, only weight gain was included as a covariate. Differences between groups were tested with log‐rank tests. SAS version 9.4 (SAS Institute Inc., Cary, NC) was used for all statistical analyses.

## 3 Results

A total of 1030 patients treated with standard platinum‐based doublet chemotherapy were included. Patients were predominantly men (70.5%) with stage IV NSCLC (Table 1). Baseline patient characteristics were generally similar across sexes, except for the presence of adenocarcinoma (more common in women) and smoking history (more common in men) (Table 1). The median age of men and women was 62 and 60 years, and the mean body mass index was 24.5 and 24.8 kg/m^2^, respectively. Most patients were current/previous smokers (94.2% men, 67.4% women).

**TABLE 1:** Key baseline demographic and clinical characteristics.

Characteristic	All (*N* = 1030)	Men (*n* = 726)	Women (*n* = 304)
Age, median (range), years	62 (34–87)	62 (34–87)	60 (34–83)
Race, *n* (%)			
Asian	172 (16.7)	126 (17.4)	46 (15.2)
Non‐Asian	857 (83.3)	600 (82.6)	257 (84.8)
BMI, mean (SD), kg/m^2^	24.6 (4.4)	24.5 (4.2)	24.8 (4.8)
Stage IV disease, *n* (%)	912 (88.5)	642 (88.4)	270 (88.8)
Adenocarcinoma, *n* (%)	380 (36.9)	232 (32.0)*	148 (48.7)
Current or previous smokers, *n* (%)	889 (86.3)	684 (94.2)*	205 (67.4)
ECOG performance status, *n* (%)			
0	358 (35.1)	245 (34.1)	113 (37.5)
1	661 (64.9)	473 (65.9)	188 (62.5)

*Note:* Percentages were calculated based on total number of patients with non‐missing data.

Abbreviations: BMI, body mass index; ECOG, Eastern Cooperative Oncology Group; SD, standard deviation.

*Significantly different from women based on chi‐square analysis (*p* < 0.0001).

**FIGURE 1:**
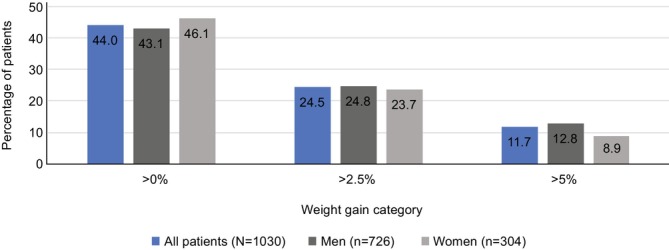
Percentage of 1030 patients with advanced non‐small cell lung cancer, including 726 men and 304 women, with any (> 0%) weight gain, > 2.5% weight gain or > 5% weight gain within the first 3 months after initiation of chemotherapy.

Overall, 453 (44.0%), 252 (24.5%) and 120 (11.7%) patients experienced weight gain from baseline to 3 months after initiating chemotherapy of > 0%, > 2.5% and > 5%, respectively. Median time to > 0%, > 2.5% and > 5% weight gain was 23, 43 and 45 days, respectively. The proportion of patients with > 0%, > 2.5% and > 5% weight gain was similar between men and women (Figure 1).

**FIGURE 2:**
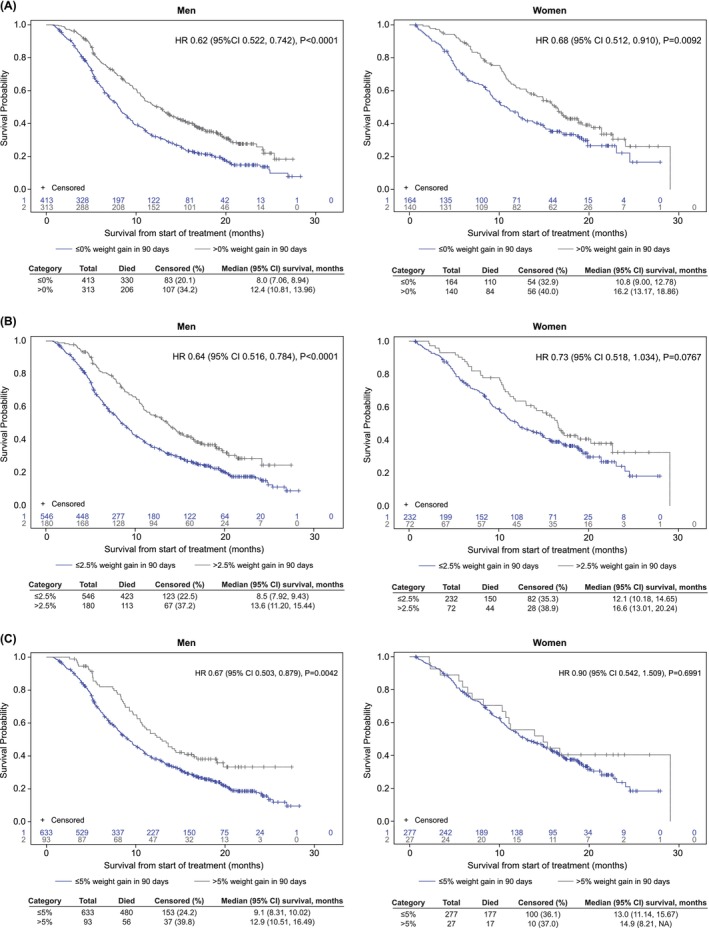
Kaplan–Meier curves of overall survival by weight gain category of > 0% vs. ≤ 0% (A), > 2.5% vs. ≤ 2.5% (B) and > 5% vs. ≤ 5% (C) in men and women with advanced non‐small cell lung cancer within the first 3 months after initiation of chemotherapy. CI, confidence interval; HR, hazard ratio, NA, not available.

Any weight gain (i.e., > 0% vs. ≤ 0%) was associated with a significantly reduced risk of death in both men (HR 0.62; 95% CI 0.522, 0.742) and women (HR 0.68; 95% CI 0.512, 0.910), with no significant interaction of weight gain by sex (*p* = 0.61). The median OS for patients with > 0% vs. ≤ 0% weight gain was 12.4 vs. 8.0 months (*p* < 0.0001) in men and 16.2 vs. 10.8 months (*p* = 0.0092) in women (Figure 2A). Similarly, weight gain of > 2.5% vs. ≤ 2.5% was associated with a significantly reduced risk of death in men (HR 0.64; 95% CI 0.516, 0.784) and a risk reduction that approached significance in women (HR 0.73; 95% CI 0.518, 1.034); weight gain by sex interaction was not significant (*p* = 0.43). The median OS for patients with > 2.5% vs. ≤ 2.5% weight gain was 13.6 vs. 8.5 months (*p* < 0.0001) in men and 16.6 vs. 12.1 months (*p* = 0.0767) in women (Figure 2B). Weight gain of > 5% vs. ≤ 5% was associated with a significantly reduced risk of death in men (HR 0.67; 95% CI 0.503, 0.879), but not in women (HR 0.90; 95% CI 0.542, 1.509). Despite these differences, the overall interaction of weight gain by sex was not significant (*p* = 0.37). The median OS for patients with > 5% vs. ≤ 5% weight gain was 12.9 vs. 9.1 months (*p* = 0.0042) in men and 14.9 vs. 13.0 months (*p* = 0.70) in women (Figure 2C).

## 4 Discussion

We observed that any weight gain during treatment of advanced NSCLC was associated with a significantly reduced risk of death in men and women. The observation that men with advanced NSCLC experienced a significantly greater loss of muscle mass during chemotherapy [11] prompted this post hoc analysis of the relationship between weight gain and OS in men vs. women with advanced NSCLC receiving standard‐of‐care platinum doublets. Consistent with previous reports in patients with NSCLC [12], men had inferior OS. Similar proportions of men and women demonstrated that weight gain during the 3 months after initiating treatment, and the occurrence of weight gain was associated with a significantly reduced risk of death in both men and women, even with baseline differences in the frequency of adenocarcinoma and smoking history between men and women.

Our study used percent weight gain cut‐offs of > 0%, > 2.5% and > 5% and categorized patients based on whether they achieved these levels of weight gain at any time during the 3‐month study period. Weight gains of > 0%, > 2.5% and > 5% generally appeared to have similar associations with increased survival. The observed lack of a significant association between > 5% weight gain and survival in women may be due to the small sample size (*n* = 27).

The study had limitations. It was not possible to evaluate causality from the study data, and the analysis did not assess whether weight gain had to be sustained to show a relationship with survival. Also, we did not evaluate whether weight gain was associated with improved survival compared to weight stabilization. Diurnal changes in weight are expected, and weight changes up to 2.4% have been classified previously as ‘stable weight.’ [13].

Collectively, our results suggest that any weight gain during treatment of advanced NSCLC was associated with a significantly reduced risk of death in both men and women.

## Funding

This study was sponsored by Pfizer. Medical writing support was provided by Diane Hoffman, PhD, of Engage Scientific Solutions, and was funded by Pfizer.

## Ethics Statement

The authors certify that they comply with the ethical guidelines for publishing in the Journal of Cachexia, Sarcopenia and Muscle [14]. The clinical studies were conducted in compliance with the ethical principles originating in or derived from the Declaration of Helsinki and in compliance with all International Conference on Harmonisation Good Clinical Practice Guidelines. All participants provided written informed consent.

## Conflicts of Interest

E.J.R. is a member of the scientific advisory board for Pfizer, Napo Pharmaceuticals and Actimed Therapeutics; consultant for Veloxis Therapeutics, Dexcel Pharma and Ryvu Therapeutics; receives research funding from Napo Pharmaceuticals, Veloxis Therapeutics, and Pfizer (all to institution); and is an expert witness for Heron Therapeutics. F.J.F. receives research support from Pfizer, serves as a consultant for BD Biosciences and serves as a consultant and speaker for Boston Scientific. R.Y. and L.T. are full‐time employees and shareholders of Pfizer. P.D.B. has received honoraria from Helsinn Therapeutics and Pfizer for participation in advisory boards.

## Data Availability

Upon request, and subject to review, Pfizer will provide the data that support the findings of this study. Subject to certain criteria, conditions and exceptions, Pfizer may also provide access to the related individual de‐identified participant data. See https://www.pfizer.com/science/clinical‐trials/trial‐data‐and‐results for more information.

## References

[1] A. R. Bruggeman , A. H. Kamal , T. W. LeBlanc , J. D. Ma , V. E. Baracos , and E. J. Roeland , “Cancer Cachexia: Beyond Weight Loss,” Journal of Oncology Practice 12 (2016): 1163–1171. 27858548 10.1200/JOP.2016.016832

[2] K. Fearon , F. Strasser , S. D. Anker , et al., “Definition and Classification of Cancer Cachexia: An International Consensus,” Lancet Oncology 12 (2011): 489–495. 21296615 10.1016/S1470-2045(10)70218-7

[3] E. J. Roeland , K. Bohlke , V. E. Baracos , et al., “Management of Cancer Cachexia: ASCO Guideline,” Journal of Clinical Oncology 38 (2020): 2438–2453. 32432946 10.1200/JCO.20.00611

[4] D. J. Sher , B. T. Gielda , M. J. Liptay , et al., “Prognostic Significance of Weight Gain During Definitive Chemoradiotherapy for Locally Advanced Non‐Small‐Cell Lung Cancer,” Clinical Lung Cancer 14 (2013): 370–375. 23260389 10.1016/j.cllc.2012.10.009

[5] J. D. Patel , J. R. Pereira , J. Chen , et al., “Relationship Between Efficacy Outcomes and Weight Gain During Treatment of Advanced, Non‐Squamous, Non‐Small‐Cell Lung Cancer Patients,” Annals of Oncology 27 (2016): 1612–1619. 27217544 10.1093/annonc/mdw211

[6] B. T. Gielda , P. Mehta , A. Khan , et al., “Weight Gain in Advanced Non–Small‐Cell Lung Cancer Patients During Treatment With Split‐Course Concurrent Chemoradiotherapy Is Associated With Superior Survival,” International Journal of Radiation Oncology, Biology, Physics 81 (2011): 985–991. 20932684 10.1016/j.ijrobp.2010.06.059

[7] E. Topkan , C. Parlak , and U. Selek , “Impact of Weight Change During the Course of Concurrent Chemoradiation Therapy on Outcomes in Stage IIIB Non‐Small Cell Lung Cancer Patients: Retrospective Analysis of 425 Patients,” International Journal of Radiation Oncology, Biology, Physics 87 (2013): 697–704. 24035331 10.1016/j.ijrobp.2013.07.033

[8] J. Le‐Rademacher , C. Lopez , E. Wolfe , et al., “Weight Loss Over Time and Survival: A Landmark Analysis of 1000+ Prospectively Treated and Monitored Lung Cancer Patients,” Journal of Cachexia, Sarcopenia and Muscle 11 (2020): 1501–1508. 32940014 10.1002/jcsm.12625 PMC7749536

[9] E. J. Roeland , F. J. Fintelmann , F. Hilton , et al., “The Relationship Between Weight Gain During Chemotherapy and Outcomes in Patients With Advanced Non‐Small Cell Lung Cancer,” Journal of Cachexia, Sarcopenia and Muscle 15 (2024): 1030–1040. 10.1002/jcsm.13426 PMC11154746 38468440

[10] M. M. Oken , R. H. Creech , D. C. Tormey , et al., “Toxicity and Response Criteria of the Eastern Cooperative Oncology Group,” American Journal of Clinical Oncology 5 (1982): 649–655. 7165009

[11] M. K. Jang , C. Park , S. Hong , H. Li , E. Rhee , and A. Z. Doorenbos , “Skeletal Muscle Mass Change During Chemotherapy: A Systematic Review and Meta‐Analysis,” Anticancer Research 40 (2020): 2409–2418. 32366384 10.21873/anticanres.14210

[12] A. L. Visbal , B. A. Williams , F. C. Nichols, 3rd , et al., “Gender Differences in Non–Small‐Cell Lung Cancer Survival: An Analysis of 4,618 Patients Diagnosed Between 1997 and 2002,” Annals of Thoracic Surgery 78 (2004): 209–215 discussion 215. 15223430 10.1016/j.athoracsur.2003.11.021

[13] L. Martin , P. Senesse , I. Gioulbasanis , et al., “Diagnostic Criteria for the Classification of Cancer‐Associated Weight Loss,” Journal of Clinical Oncology 33 (2015): 90–99. 25422490 10.1200/JCO.2014.56.1894

[14] S. von Haehling , A. J. S. Coats , and S. D. Anker , “Ethical Guidelines for Publishing in the Journal of Cachexia, Sarcopenia and Muscle: Update 2023,” Journal of Cachexia, Sarcopenia and Muscle 14 (2023): 2981–2983. 38148513 10.1002/jcsm.13420 PMC10751405

